# Giant Hydatid Cyst within a Congenital Cystic Adenomatoid Malformation of the Lung

**Published:** 2013-05-09

**Authors:** Yogesh Kumar Sarin, Shalini Sinha, Anju Romina Bhalotra, Nita Khurana, Parul Sobti

**Affiliations:** Department of Paediatric Surgery, Maulana Azad Medical College New Delhi, India; Department of Paediatric Surgery, Maulana Azad Medical College New Delhi, India; Department of Anaesthesia, Maulana Azad Medical College New Delhi, India; Department of Pathology, Maulana Azad Medical College New Delhi, India; Department of Pathology, Maulana Azad Medical College New Delhi, India

**Keywords:** Hydatid cyst, Congenital cystic adenomatoid malformation, Pericystectomy

## Abstract

A case of hydatid cyst within a congenital cystic adenomatoid malformation (CCAM) of the right lower lobe of lung in an 8-year-old girl is reported. Presence of CCAM was confirmed on histopathology of the lung tissue attached to the specimen.

## INTRODUCTION

Hydatid cyst occurring in a pre-existing CCAM of the lung is extremely rare [1-3]. In this report we present one such case.

## CASE REPORT

An 8-year-old girl presented with chest pain and dyspnea for 7 months and productive cough for 3 months. There was no history of hemoptysis. Respiratory system examination revealed decreased breath sounds in right infra-axillary and infrascapular regions. She had been investigated elsewhere and the initial chest x-ray showed a 10x10 cm cyst in the right lower zone (Fig. 1). The diagnosis of hydatid cyst lung was made and a course of anti-helminthics was given that resulted in decrease of the cyst size. Contrast enhanced CT scan thorax revealed an 8.5x7.5x6.5 cm air-filled cavitary lesion with detached dependent wavy membrane in the lower lobe of right lung with collapse / consolidation of the adjacent lung parenchyma (Fig. 2). There was evidence of focal pleural calcification, pleural thickening and minimal pleural effusion on the right side. Ultrasound of the abdomen was normal. Right thoracotomy revealed dense adhesions surrounding a large hydatid cyst in the right lower lobe of lung. Pericystectomy was done. Patient had an unexpected large amount of blood loss (~1300ml) and two episodes of bradycardia intra-operatively. She was actively resuscitated with blood and blood products and electively ventilated for 48 hours postoperatively. She had multiple episodes of focal as well as generalized tonic-clonic seizures over the next 3 days which were controlled with dilantin, phenytoin and midazolam. There were no obvious neurological signs. A cranial MRI done after extubation revealed bilateral hippocampal infarcts with edema and a small infarct in the cerebellum. Her seizures then stopped and there was no amnesia. 

Histopathological examination of the specimen was consistent with hydatid cyst. Fibrosis and mixed acute and chronic inflammation, which was extending into the lung parenchyma, were demonstrated. In addition, there were features of CCAM type 2 in the adjacent lung parenchyma (Fig. 3a-d). Postoperative chest x-ray showed consolidation in the right lower zone with a fibrotic opacity in the right middle zone. Postoperative CT scan chest revealed opacity in the right lower lobe with the appearance of CCAM. Right lower lobectomy was done after 2 months.

**Figure:**
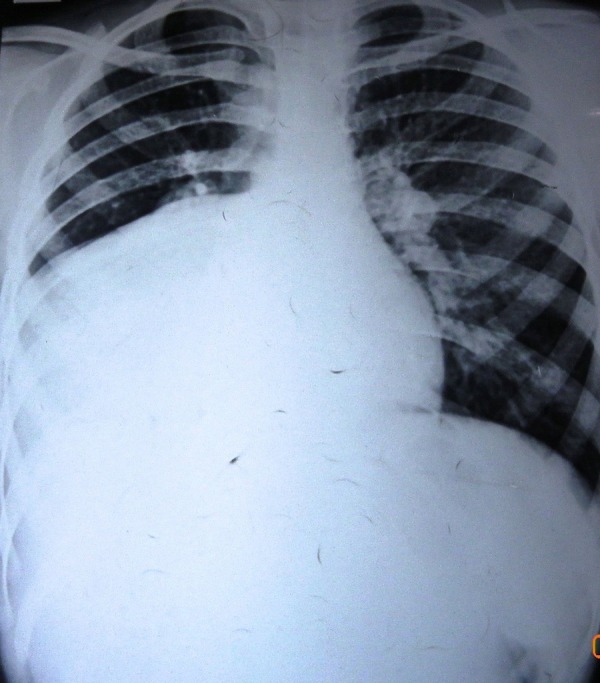
Figure 1: Plain Radiograph of the chest showing a 10 x 10cm opacity in the right lower zone of lung.

**Figure:**
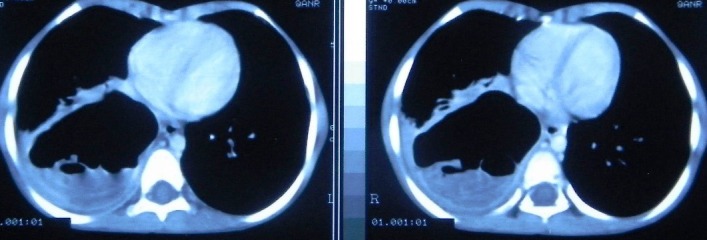
Figure 2: CT thorax showing an 8.5 x 7.5 x 6.5 cm air-filled cavitary lesion with detached dependent wavy membrane in the right lower zone of lung consistent with hydatid cyst.

**Figure:**
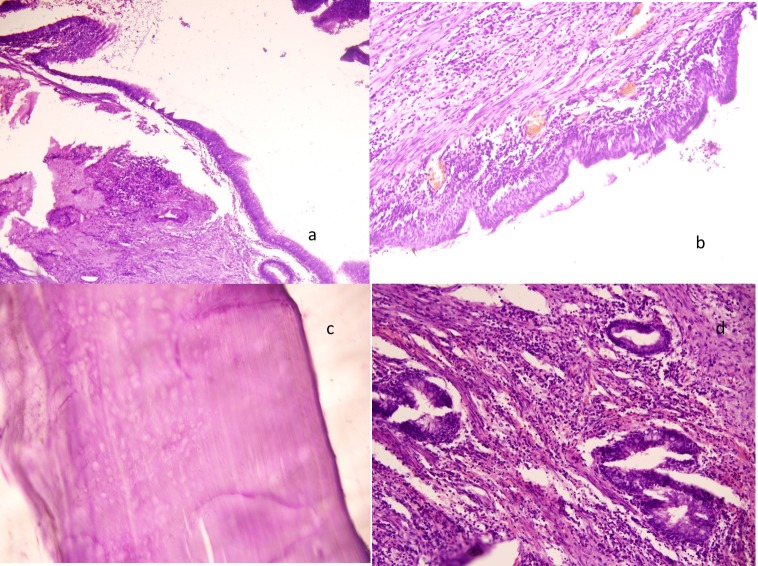
Figure 3 (a): Photomicrograph showing a large cyst lined by columnar epithelium with small glandular structures in the inflamed wall (H/E x 100), (b): cyst lined by tall columnar epithelium (H/E x 250), (c): laminated wall of the hydatid cyst (H/E x 400), (d): dense inflammation in the pericyst with entrapped adenoid structures lined by tall columnar epithelium consistent with CCAM type 2 (H/E x 400).

## DISCUSSION

CCAM is rare entity. It represents about 15% of all congenital pulmonary malformations [1]. Interestingly, pulmonary echinococcosis has been occasionally misdiagnosed as CCAM preoperatively [2].The co-existence of these two infrequent conditions is therefore intriguing. A literature search revealed a case of hydatidosis within a CCAM [3]. In this case report, the author described a 12-year-old boy with a communicating ruptured hydatid associated with consolidation of the involved right lower lobe of lung. Lobectomy was done and the histopathology confirmed the underlying CCAM in that case. In our case too, the confirmation of underlying CCAM was done only after histopathological examination of the excised specimen. The characteristic imaging features of CCAM were overshadowed by the presence of hydatid cyst and the lung parenchyma adjacent to the hydatid cyst was thought to be affected with consolidation. The sudden intraoperative blood loss with hypotension and bradycardia in this patient led to a mild form of hypoxic ischemic encephalopathy (HIE), resulting in hippocampal infarction (silent stroke). This condition has been described whenever there is ischemia lasting for more than 4-5 minutes as the hippocampal pyramidal cells are highly vulnerable [4]. Bilateral hippocampal damage is known to cause Korsakoff’s amnesia which our patient did not have. The underlying undiagnosed CCAM is likely to be the cause for the unexpected blood loss during pericystectomy.

## Footnotes

**Source of Support:** Nil

**Conflict of Interest:** None declared

